# Rituximab response in follicular lymphoma: contributions from KIR 2DS1 and HLA-C

**DOI:** 10.1186/2051-1426-1-S1-P243

**Published:** 2013-11-07

**Authors:** A K Erbe, Wei Wang, B Grzywacz, E A Ranheim, J A Hank, K Kim, L Carmichael, S Seo, E A Mendonca, Y Song, F Hong, R D Gascoyne, E Paietta, S H Horning, B Kahl, P M Sondel

**Affiliations:** 1 University of Wisconsin, Madison, WI, USA; 2 Dana-Farber Cancer, Boston, MA, USA; 3 British Columbia Cancer Agency, Vancouver, BC, Canada; 4 Montefiore Medical Center North Campus, Bronx, NY, USA; 5 Genentech, Inc., South San Francisco, CA, USA

## 

Immunotherapeutic response in cancer patients, specifically those attributed to antibody-dependent cellular cytotoxicity (ADCC), may be variable dependent upon an individual's genotype. A recent Eastern Cooperative Oncology Group (ECOG) Phase III clinical trial, E4402, sought to optimize therapeutic administration of single agent rituximab (RTX) in patients with low tumor burden follicular lymphoma by investigating two RTX maintenance strategies: Arm A (RTX given "as needed") or Arm B (continuous RTX regimen). We analyzed the DNA from these patients to determine if KIR/KIR-ligand genotype had an effect on patient response to RTX. Of several parameters assessed, the activating KIR2DS1 gene and its cognate ligand, HLA-C2, were found to yield significant associations with clinical outcome. Venstrom et al. (NEJM-2012) found in an allogeneic setting that patients receiving a hematopoietic stem cell transplant from donors positive for KIR2DS1 (2DS1+) had a lower rate of relapse than from 2DS1 negative (2DS1-) donors, and this difference was further influenced by HLA-C. Specifically, for 2DS1+ donors, the HLA-C1 positive (C1+) recipients [homozygote (C1C1) or heterozygote (C1C2)] fared better than recipients homozygous for HLA-C2 (C2C2).

In this study, we observed a similar finding, where 2DS1+ patients had a trend towards better response (tumor shrinkage) if they were C1+ rather than C2C2 (p=0.062), as well as a longer time to RTX-failure (TTRF) (p<0.001). Furthermore, we noted a trend towards a longer TTRF in patients that were C1+ vs. those that were C2C2, regardless of KIR status (p=0.071). Interestingly, unlike Venstrom et al., we found that C2C2 patients had improved tumor shrinkage (p=0.039) and improved TTRF (p=0.023) if they were 2DS1- vs. 2DS1+. These results are consistent with hyporesponsive NK cells in 2DS1+/C2C2 patients, as shown by Pittari et al. [JI-2013], such that 2DS1+ NK cells from C2C2 allogeneic donors were desensitized compared to those from C1+ donors. This is analogous to studies where the presence of an activating receptor and its cognate ligand resulted in reduced responsiveness of NK cells. Our data are concordant with previous findings in allogeneic settings. Our results suggest that C2 homozygosity may contribute to NK cell hyporesponsiveness in a 2DS1-dependent manner, even in an autologous immunotherapy setting.

